# Characteristics of pneumoconiosis in Zhejiang Province, China from 2006 to 2020: a descriptive study

**DOI:** 10.1186/s12889-023-15277-8

**Published:** 2023-02-22

**Authors:** Fang Wei, Panqi Xue, Lifang Zhou, Xinglin Fang, Yixin Zhang, Yong Hu, Hua Zou, Xiaoming Lou

**Affiliations:** 1 Occupational Health and Radiation Protection Institute, Zhejiang Provincial Center for Disease Control and Prevention, Hangzhou, China; 2 School of Medicine, Hangzhou Normal University, Hangzhou, China

**Keywords:** Pneumoconiosis, Occupational disease, Incidence pattern, Temporal trends, Epidemiology

## Abstract

**Background:**

Pneumoconiosis is the most prevalent occupational disease and displays different patterns in each province of China. Clarifying specific incidence patterns and temporal trends in Zhejiang Province can help provide valuable information on the prevention of pneumoconiosis.

**Methods:**

Annual reports of pneumoconiosis for Zhejiang Province from 2006 to 2020 were extracted from the National Occupational Disease and Occupational Health Information Monitoring System. The information of cases included regions, diagnosis ages, genders, exposure durations, pneumoconiosis categories and stages, the first year of exposure, enterprise industries, scales and ownerships.

**Results:**

Totally 6037 new cases of pneumoconiosis were reported between 2006 and 2020, which increased at first and then gradually declined since 2013. Among all pneumoconiosis cases, silicosis accounted for the majority (72.17%). Most of the cases occurred in small-scale and domestic-funded enterprises, which accounted for 71.75% and 96.97%, respectively. When analyzing the industry distribution, the cases were mainly concentrated in mining (37.12%), manufacturing (31.11%) and ‘public administration and social organization’ (23.94%) industry. The average diagnosis age among the pneumoconiosis cases was 55.44 years, and the median exposure duration was 11.00 years. Significantly older diagnosis age and longer exposure duration were found in females, coal workers’ pneumoconiosis cases, cases with higher stages, cases with the first year of dust exposure earlier and cases from large-scale companies. In regional distribution, the top three cities reporting the most pneumoconiosis cases in Zhejiang Province were Taizhou, Quzhou and Hangzhou.

**Conclusion:**

The current situation of pneumoconiosis in Zhejiang Province was still serious, and government should further strengthen the surveillance of occupational diseases and supervision of enterprises. Moreover, publicity and education regarding pneumoconiosis should be carried out to raise awareness of dust exposure risk and associated health consequences.

**Supplementary Information:**

The online version contains supplementary material available at 10.1186/s12889-023-15277-8.

## Background

Pneumoconiosis, including a group of progressive and irreversible occupational lung diseases, is caused by prolonged inhalation of productive mineral dusts in professional activities [1]. Pneumoconiosis has been classified into 13 categories, including silicosis, coal workers’ pneumoconiosis (CWP), welders’ pneumoconiosis, asbestosis and so on. With the progression of pneumoconiosis, dust accumulated in lungs could trigger alveoli inflammation and pulmonary fibrosis. The pathological changes progressed even after transferring away from the positions exposed to dust, which would eventually lead to irreversible lung damage and respiratory failure even death [2]. Previous studies have reported that pneumoconiosis patients were more likely to develop tuberculosis [3, 4]. Besides, a cohort study among metal mine and pottery workers in China revealed that silica exposure was associated with the elevated risk of mortality [5]. More recently, a survival analysis of 15,402 pneumoconiosis cases in China found that the average survival time of pneumoconiosis cases was 14.74 ± 9.57 years, and the mortality rate was 19.89% [6]. Pneumoconiosis is not only a severe issue related to public health, but also can impose a substantial economic burden on patients and society.

Pneumoconiosis has long been the most prevalent occupational disease, and China is one of the countries with the most dust-exposed population and pneumoconiosis cases among the world [7]. The National Health Commission of the People’s Republic of China indicated that there were more than 97,500 cases of occupational diseases reported until 2018, of which 90% were pneumoconiosis [8]. Since the discovery of pneumoconiosis in the 19th century, many types of public health measures have been enacted to counter this problem, while the number of newly diagnosed pneumoconiosis still increased on the global scale [9]. In China, strict occupational exposure limits were established, but the implementation of the standards was poor, especially in some economically backward cities [10]. It was reported that only 30% of the workers were subjected to occupational health surveillance [11], which inferred that the number of pneumoconiosis cases was greatly underestimated.

Till now, there is no effective curative treatment for pneumoconiosis, and the prevention is still the main means to reduce morbidity. Identifying the pneumoconiosis incidence patterns and temporal trends is important to help facilitate the allocation of healthcare resources rationally. Since pneumoconiosis displays different patterns in each province of China, exploring the incidence characteristics and dynamic changes of pneumoconiosis in each province is necessary to help take targeted measures to prevent and control pneumoconiosis. Zhejiang Province is a southeastern coastal province with a great number of industrial enterprises, of which a large proportion exist occupational hazards factors. In present study, we analyzed the characteristics of newly reported pneumoconiosis cases in Zhejiang Province from 2006 to 2020, which could provide valuable information on the incidence and prevention of pneumoconiosis.

## Methods

### Study areas

Zhejiang Province, where the gross domestic product (GDP) reaches 735.16 billion Yuan in 2021, is a major coastal province covering 105,500 km^2^ in the Yangtze River Delta region of China. According to the National Bureau of Statistics, the resident population of Zhejiang Province in 2021 is 65.4 million, which includes 39 million workers [12]. Zhejiang Province is rich in mineral resources, and chemical, textile, equipment manufacturing, metal products manufacturing, metal smelting and rolling processing industries are the mainstay industries in Zhejiang Province. While providing a labor market and promoting economic development, these industries also bring severe occupational hazards such as dust hazards that cannot be ignored.

### Data sources

Annual reports of pneumoconiosis for Zhejiang Province from 2006 to 2020 were extracted from the National Occupational Disease and Occupational Health Information Monitoring System. The system was constructed in 2006, which included the basic information of all confirmed occupational disease cases, such as dates of birth, genders, occupational disease categories, diagnosis dates, exposure durations and employer information. Once a worker was confirmed to have occupational pneumoconiosis by occupational disease diagnostic physicians, an occupational disease notification card will be submitted to the system. All cards uploaded to the system would be reviewed and approved by physicians in centers for disease control and prevention (CDC) at the county/district, city, provincial and national levels, which ensured the comprehensiveness and reliability of the reporting data.

### Pneumoconiosis diagnosis

Pneumoconiosis cases were identified through occupational health examination or routine surveillance. In China, radiography was an essential tool in the pneumoconiosis diagnosis. The diagnoses were performed by at least three radiologists using the “Diagnostic Criteria of Pneumoconiosis”, which was based on the occupational history and chest radiograph. The criteria classified pneumoconiosis into three stages as stage I, II and III according to the size, profusion and distribution of opacities on chest X-ray.

### Definition

On the basis of “industrial classification for national economic activities (GB/T 4754 − 2017)”, industry type was divided into 20 categories according to the main economic activities, which accounted for the most economic income or working hours. Here, we combined some industries with fewer pneumoconiosis cases into “others”, leaving other 6 major industries including mining, manufacturing, construction, ‘traffic, storage and mail business’, ‘neighborhood services and other service industry’ and ‘public administration and social organization’.

The enterprise scale was divided into large, medium, small and micro scale according to the enterprise scale standard established by the National Statistics Bureau of the People’s Republic of China. The enterprises with employees ≥ 1000 and operation revenue ≥ 400 million were defined as large-scale; The enterprises with 300 ≤ employees < 1000 and 20 ≤ operation revenue < 400 million were defined as medium-scale; The enterprises with 20 ≤ employees < 300 and 3 ≤ operation revenue < 20 million were defined as small-scale; The enterprises with employees < 20 and operation revenue < 3 million were defined as micro-scale. For some enterprises with an unknown number of employees and operation revenue, the enterprise scale was classified as unknown.

Enterprise ownership was divided into 3 categories: Domestic-funded, Hong Kong/Macao/Taiwan- funded and Foreign-funded, which was based on the ownership of the major capital.

### Statistical analyses

Continuous variables were described as mean ± standard deviation (SD) or median (interquartile range, IQR) according to normality tested by QQ plot, and categorical variables were described as frequency and percentages. ANOVA test or Kruskal-Wallis tests and Chi-square tests were used to compare the differences between groups for continuous variables and categorical variables, respectively. Tukey HSD and Nemenyi test were used for the post hoc multiple comparisons.

All analyses were carried out using R software 4.1.2. Two-tailed *P* < 0.05 was considered statistically significant.

## Results

**Fig. 1:**
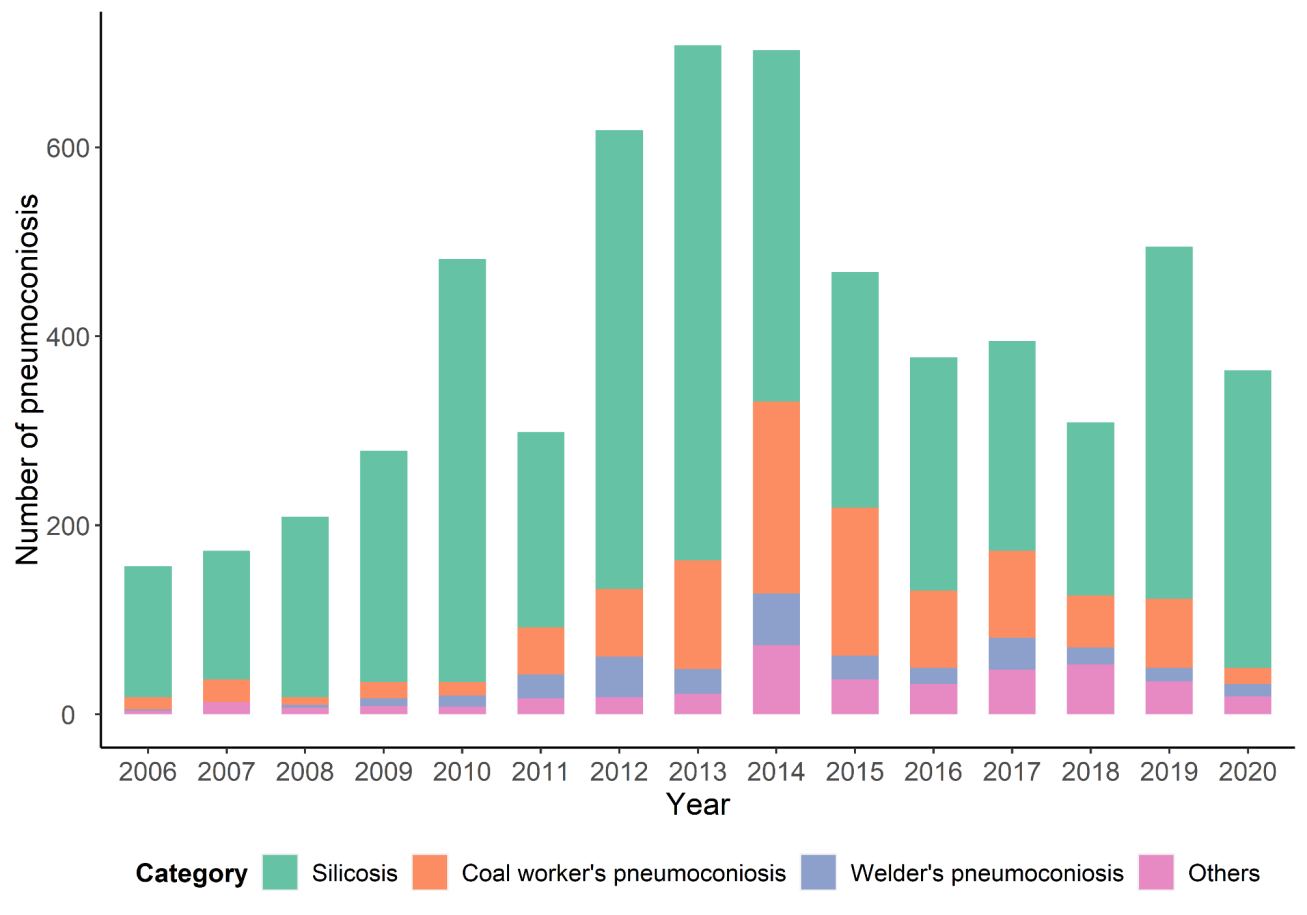
The number of pneumoconiosis cases reported in Zhejiang Province from 2006 to 2020

From 2006 to 2020, there were totally 6037 new cases of pneumoconiosis reported in the Zhejiang Province. Since 2006, the number of cases showed an increasing trend at first, reaching the highest in 2013 with 708 cases, and then displayed an overall declining trend, which was shown in Fig. 1. Silicosis was the most prevalent category accounting for 72.17%, followed by CWP (16.43%), and welders’ pneumoconiosis (4.87%). The trend of CWP and welders’ pneumoconiosis showed rising first and then slowly declining with fluctuation, while for silicosis there was an upward trend since 2019 (**Figure S1**).

**Fig. 2:**
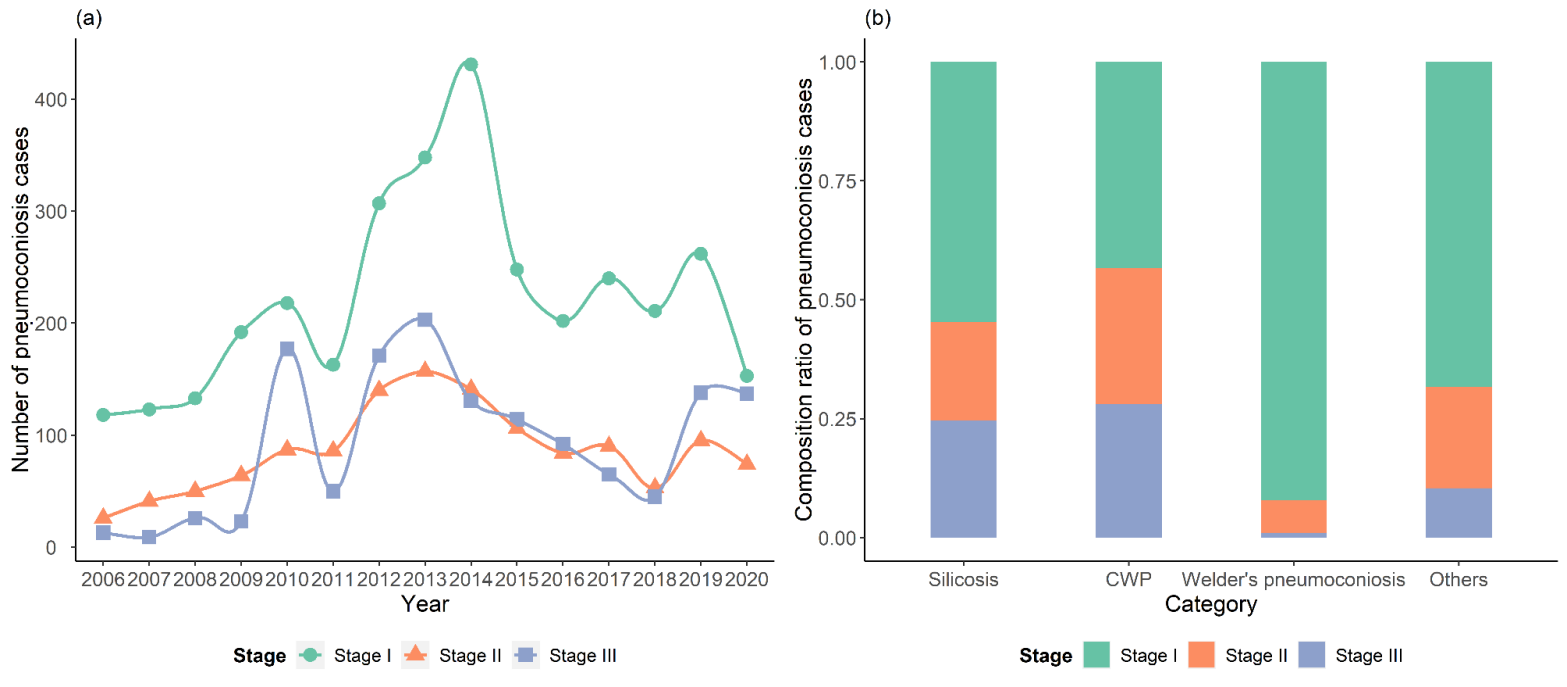
The stage of pneumoconiosis cases reported in Zhejiang Province from 2006 to 2020

From 2006 to 2020, there were 3349, 1294 and 1394 pneumoconiosis cases of Stage I, II and III, respectively, with stage I accounting for approximately 55.47% of the total. As shown in Fig. 2a, stage I, II and III all showed a general tendency of rising from 2006 to 2013, and then obviously decreased. When considering the stage distribution for different categories of pneumoconiosis, welders’ pneumoconiosis was mainly in stage I (92.18%), which was shown in Fig. 2b.

As shown in Table 1, cumulative pneumoconiosis cases mainly occurred in the mining industry, manufacturing industry, and ‘public administration and social organization’, whose number were 2241 (37.12%), 1878 (31.11%) and 1445 (23.94%). For silicosis and welders’ pneumoconiosis, the industries with the largest number were mining industry and manufacturing industry, respectively. And 68.95% of CWP cases were distributed in mining industry, followed by ‘public administration and social organization’ which accounting for 24.29%. In terms of enterprise ownership, most of the cases were reported in small-scale enterprises with 4332 cases (71.75%). About 96.97% pneumoconiosis cases were from domestic-funded enterprises, and those from Foreign-funded and Hong Kong/Macao/Taiwan-funded accounted for 1.89% and 1.14%, respectively.

**Table 1:** The industry type, enterprise scale and ownership type distribution of pneumoconiosis cases in Zhejiang Province from 2006 to 2020

Characteristics	Total	Silicosis	CWP	Welders’ pneumoconiosis	Others
**Industry type**					
Mining industry	2241 (37.12%)	1543 (35.41%)	684 (68.95%)	3 (1.02%)	11 (2.79%)
Manufacturing industry	1878 (31.11%)	1249 (28.67%)	52 (5.24%)	275 (93.54%)	302 (76.65%)
Construction industry	162 (2.68%)	141 (3.24%)	6 (0.60%)	10 (3.40%)	5 (1.27%)
Traffic, storage and mail business	127 (2.10%)	109 (2.50%)	0 (0.00%)	0 (0.00%)	18 (4.57%)
Neighborhood services and other service industry	74 (1.23%)	71 (1.63%)	2 (0.20%)	0 (0.00%)	1 (0.25%)
Public administration and social organization	1445 (23.94%)	1153 (26.46%)	241 (24.29%)	2 (0.68%)	49 (12.44%)
Others	110 (1.82%)	91 (2.09%)	7 (0.71%)	4 (1.36%)	8 (2.03%)
**Enterprise scale**					
Large	332 (5.50%)	142 (3.26%)	145 (14.62%)	32 (10.88%)	13 (3.30%)
Medium	840 (13.92%)	625 (14.34%)	65 (6.55%)	88 (29.93%)	62 (15.74%)
Small	4332 (71.75%)	3088 (70.87%)	757 (76.31%)	172 (58.50%)	315 (79.95%)
Micro	25 (0.41%)	18 (0.41%)	1 (0.10%)	2 (0.68%)	4 (1.02%)
Unknown	508 (8.42%)	484 (11.11%)	24 (2.42%)	0 (0.00%)	0 (0.00%)
**Enterprise ownership**					
Domestic-funded	5854 (96.97%)	4241 (97.34%)	988 (99.60%)	257 (87.41%)	368 (93.40%)
Hong Kong/Macao/Taiwan- funded	69 (1.14%)	48 (1.10%)	1 (0.10%)	12 (4.08%)	8 (2.03%)
Foreign-funded	114 (1.89%)	68 (1.56%)	3 (0.30%)	25 (8.50%)	18 (4.57%)

The average diagnosis age among the cases was 55.44 years, and the median exposure duration was 11.00 years. **Figure S2** was drawn to intuitively reflect the distribution characteristics and changing trend, which indicated that diagnosis age and exposure duration both showed a rising trend with little fluctuation. As summarized in Table 2, significantly older diagnosis age and longer exposure duration were found in females and cases with higher pneumoconiosis stages. Compared with CWP, silicosis and welders’ pneumoconiosis cases had younger diagnosis age and shorter exposure duration, with the post hoc comparisons shown in **Table S2**. Furthermore, the average age and median exposure duration decreased along with the increase of the first year of dust exposure. When considering industry type, the manufacturing industry seemed to have the youngest diagnosis age and other industry type to have the shortest exposure duration. Regarding enterprise size, cases from large-scale companies had the oldest diagnosis age and longest exposure duration, while micro-scale enterprises had the youngest diagnosis age and shortest exposure duration.

**Table 2:** Diagnosis age and exposure duration of pneumoconiosis cases in Zhejiang Province from 2006 to 2020

Characteristics	Pneumoconiosis cases, n (%)	Diagnosis age, year	Exposure duration, year
**Total**	6037 (100.00%)	55.44 ± 10.14	11.00 (6.50, 18.00)
**Gender**			
Male	5865 (97.15%)	55.30 ± 10.07	11.00 (6.42, 18.00)
Female	172 (2.85%)	60.19 ± 11.30	12.04 (9.25, 20.00)
*P* value	-	< 0.001	0.003
**Category**			
Silicosis	4357 (72.17%)	55.43 ± 9.82	11.00 (6.25, 18.50)
CWP	992 (16.43%)	59.58 ± 9.10	12.00 (8.00, 18.00)
Welders’ pneumoconiosis	294 (4.87%)	43.31 ± 7.22	9.00 (6.00, 12.08)
Others	394 (6.53%)	54.28 ± 10.41	10.33 (6.60, 16.54)
*P* value	-	< 0.001	< 0.001
**Stage**			
Stage I	3349 (55.47%)	54.99 ± 10.83	10.67 (6.00, 18.25)
Stage II	1294 (21.43%)	55.20 ± 9.54	11.08 (6.92, 18.00)
Stage III	1394 (23.09%)	56.75 ± 8.78	11.75 (7.92, 17.42)
*P* value	-	< 0.001	0.011
**First year of dust exposure**			
<1980	2354 (38.99%)	63.10 ± 7.82	13.00 (7.00, 20.56)
1980-	1164 (19.28%)	54.81 ± 7.41	15.00 (10.00, 20.27)
1990-	927 (15.36%)	50.04 ± 8.14	13.33 (9.00, 18.29)
2000-	1262 (20.90%)	47.55 ± 7.70	8.33 (5.58, 11.00)
2010-	330 (5.47%)	48.40 ± 6.99	4.25 (2.92, 6.00)
*P* value	-	< 0.001	< 0.001
**Industry type**			
Mining industry	2241 (37.12%)	55.63 ± 9.17	13.17 (8.00, 20.08)
Manufacturing industry	1878 (31.11%)	49.73 ± 9.50	10.00 (6.08, 15.17)
Construction industry	162 (2.68%)	54.39 ± 9.54	10.00 (5.08, 18.50)
Traffic, storage and mail business	127(2.10%)	63.20 ± 10.39	8.67 (4.00, 29.13)
Neighborhood services and other service industry	74 (1.23%)	63.28 ± 7.02	11.08 (8.08, 14.08)
Public administration and social organization	1445 (23.94%)	61.19 ± 7.74	10.92 (6.00, 17.67)
Others	110 (1.82%)	61.02 ± 13.34	6.45 (3.50, 10.56)
*P* value	-	< 0.001	< 0.001
**Enterprise scale**			
Large	332 (5.50%)	57.97 ± 13.50	17.75 (9.06, 25.85)
Medium	840 (13.92%)	51.73 ± 10.55	11.25 (6.67, 19.79)
Small	4332 (71.75%)	55.91 ± 9.56	11.00 (6.67, 17.00)
Micro	25 (0.41%)	49.70 ± 8.79	7.73 (3.58, 9.08)
Unknown	508 (8.42%)	56.24 ± 10.27	11.25 (4.92, 18.94)
*P* value	-	< 0.001	< 0.001

**Fig. 3:**
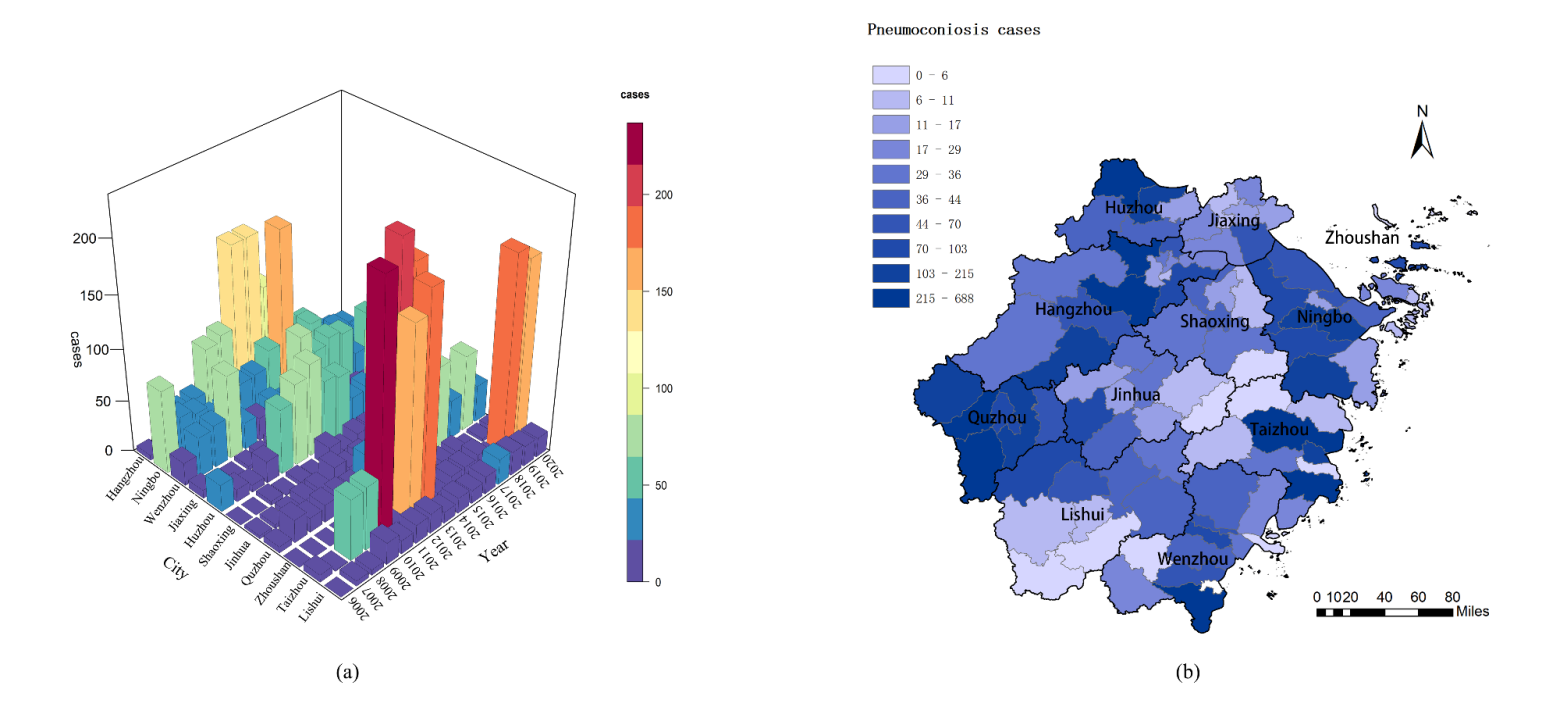
The temporal and regional distribution of pneumoconiosis cases reported in Zhejiang Province from 2006 to 2020

As depicted in Fig. 3a, Ningbo was the city reported the most cases (N = 80, 50.96%) in 2006, while in 2020 it only reported 27 cases (7.42%) with a sharply decreasing trend. For Taizhou and Quzhou, where reported 7 (4.46%) and 6 (3.82%) cases in 2006, the number of new cases rose to 178 (48.90%) and 36 (9.89%) cases in 2020. It is worth noting that even more than 200 cases were reported in some years in Taizhou and Quzhou, and the detailed information was listed in **Table S1**. Figure 3b depicted the regional distribution of newly diagnosed pneumoconiosis cases. From 2006 to 2020, the top three cities reporting the most pneumoconiosis cases in Zhejiang Province were Taizhou with 1180 cases (19.55%), Quzhou with 956 cases (15.84%) and Hangzhou with 927 cases (15.36%).

## Discussion

Discerning the incidence patterns and temporal trends is critical to effectively enhancing pneumoconiosis prevention. If we only focused on the total number of pneumoconiosis cases as a whole, the actual trends and characteristics may be neglected, leading to idle work to prevent pneumoconiosis. In present study, we analyzed the reporting pneumoconiosis data from 2006 to 2020 in Zhejiang Province to explore the incidence patterns. Totally 6037 new cases of pneumoconiosis were reported in the Zhejiang Province during this period, which increased at first and then gradually declined since 2013. The average diagnosis age among the pneumoconiosis cases was 55.44 years, and the median exposure duration was 11.00 years. Compared with other cities in Zhejiang Province, the pneumoconiosis incidence was relatively higher in Taizhou, Quzhou and Hangzhou.

The Global Burden of Disease studies reported that the incidence of pneumoconiosis worldwide showed a downward trend since 2015 [13, 14]. In Zhejiang Province, we found a similar trend that reached the highest in 2013 and then displayed an overall declining trend. Some explanations may account for this phenomenon. Firstly, China promulgated the Occupational Disease Prevention and Control Law in 2001 and constructed the National Occupational Disease and Occupational Health Information Monitoring System in 2006, which gradually strengthen the surveillance of occupational diseases. In addition, the workers’ awareness of protecting their health and legitimate rights gradually increased, and more workers requested occupational diseases diagnosis after the onset of symptoms, leading to the increasing trend since 2006. Meanwhile, enterprises began to strengthen the management of occupational health with the popularization of the awareness of occupational diseases prevention [15]. Considering the relatively long incubation period of pneumoconiosis [16], the yearly number of pneumoconiosis cases gradually declined after 2013.

In present study, silicosis accounted for the majority of the pneumoconiosis cases with 72.17%, and the proportion was close to Jiangsu Province (72.9%) [17]. Nevertheless, the Chinese Center for Disease Control and Prevention revealed that the rate of silicosis was 41.85% across China in 2010 [18]. Besides, Gansu Province and Hunan Province also reported that silicosis accounted for 55.71% and 50.69%, respectively [19, 20], which was significantly lower than ours. This indicated that the situation of silicosis in Zhejiang Province was more serious than in some other regions in China. For CWP, which accounted for 16.43% of all pneumoconiosis cases, the proportion was obviously lower than 72.02% reported in a city in Jiangxi Province [21]. These can be caused by the relatively smaller proportion of the coal mining industry in Zhejiang Province compared with Jiangxi.

There were almost half of the cases found stage II or stage III at their first pneumoconiosis diagnosis. These cases were usually exposed to high-concentration dust with severe hazards, suggesting that government should strengthen the occupational health management of enterprises to improve the working environment. Besides, this phenomenon also indicated that the regular occupational health examination was not implemented strictly, resulting in missed opportunities to detect pneumoconiosis at its early stages. Furthermore, many cases with severe dust exposure and long exposure duration of those bankrupt enterprises were diagnosed after the promulgation of the Law of Occupational Disease Prevention and Treatment in 2001, when their pneumoconiosis has already progressed to stage II or stage III [16]. All of the reasons above may contribute to the high proportion of stage II and stage III cases at first diagnosis.

The cases of silicosis in Zhejiang Province were mainly concentrated in mining (35.41%), manufacturing (28.67%) and ‘public administration and social organization’ (26.46%) industry. Silicosis was historically regarded as a disease of miners, while recent decades the silicosis incidence in manufacturing emerging due to the failure to recognize and control the occupational silica exposure risk in modern manufacturing industry [22]. As for the industry distribution of CWP, coal miners were always exposed to a high concentration of coal dust and then led to the high incidence of CWP [23], which resulted in CWP mainly distributing in the mining industry (68.95%). In addition, workers of those collapsed-enterprises were organized by the government for pneumoconiosis diagnosis, which was the reason for the high proportion (23.94%) of ‘public administration and social organization’ in the distribution of pneumoconiosis cases by industry.

In agreement with previous studies suggesting that a large proportion of cases were distributed in small-scale enterprises [17, 24], most of the pneumoconiosis cases in Zhejiang Province were reported in small enterprises, which accounted for 71.75% of all cases. One of the important reasons should not be ignored was that small-scale enterprises were the main enterprise scale in Zhejiang Province. According to the report of the Zhejiang Provincial Bureau of Statistics, by the end of 2020, there were 40,275 small enterprises in Zhejiang province, accounting for 83.98% of all scale enterprises [25]. Moreover, most small-scale enterprises had insufficient attention to occupational diseases, and they always had poor working conditions without basic occupational protection or personal protective equipment [24]. Especially for those migrant workers, who were always without self-protection awareness, they were more likely to be employed in dangerous positions in small-scale enterprises, and this may be another part of the explanation. Thus, the regulator should further strengthen the supervision of enterprises, especially small and micro-scale enterprises.

Considering the enterprise ownership, there were significantly more pneumoconiosis cases in domestic-funded enterprises compared with those foreign-funded or Hong Kong/Macao/Taiwan- funded enterprises. On the one hand, domestic-funded enterprises were the most common enterprise ownership in Zhejiang Province with a large base of dust workers, therefore the number of pneumoconiosis cases would correspondingly be larger. On the other hand, as some literature reported, domestic-funded enterprises always do better in occupational diseases prevention and control [17], where they detect and report patients in time.

The data in present study indicated that diagnosis age and exposure duration both showed a rising trend with little fluctuation, which suggested to some extent that the prevention and treatment of pneumoconiosis in recent years has made some progress. Males had younger diagnosis age and relatively shorter exposure duration than females, which can be partly explained by the preference of male workers in frontline jobs with severe dust exposure. Compared with coal workers’ pneumoconiosis, welding worker’s pneumoconiosis and silicosis had lower age and shorter exposure duration, indicating that welding dust and silicon dust were more harmful. And there was also literature reported that silicosis was the most common and harmful pneumoconiosis [26]. The average diagnosis age and exposure duration increased with the stage upgrade, indicating that older age and longer exposure duration were both risk factors for pneumoconiosis developing and progressing. When considering first year of exposure, the results suggested that the age and exposure duration decreased along with the increase of the first year of dust exposure, which was the same as the findings of another study conducted in China [24]. As for enterprise scale, cases from micro-scale enterprises had the youngest diagnosis ages and shortest exposure duration, while those from large-scale enterprises had the oldest diagnosis ages and longest exposure duration, which was in agreement with that reported in a research literature in Hebei [24]. This partly resulted from the poor working conditions in micro-scale enterprises, and the dust workers in small enterprises were always unaware of the potential occupational hazards due to their low educational level.

Our study presented that the top three cities reporting the most pneumoconiosis cases in Zhejiang Province were Taizhou, Quzhou and Hangzhou. This can be attributable to the industrial structure, population size and the supervision of occupational health in each city. For Taizhou and Quzhou, where there were many returned migrant workers or workers from closed-down enterprises, most of the pneumoconiosis cases were unable to confirm their labor relationship and were diagnosed by the civil affairs departments of the local government [16]. Besides, Hangzhou has 11.96 million population and 1.48 million employees in 2020 according to the Statistical Yearbook of Zhejiang Province [25], which was much higher than other cities in Zhejiang Province. The large base of workers in Hangzhou led to the relatively high number of dust workers and pneumoconiosis cases.

As we all known, high-quality occupational disease monitoring and supervision data are critical to the estimation of disease burden, which could help policy-makers to promulgate targeted policies. The data used in present study was collected from the National Occupational Disease and Occupational Health Information Monitoring System, which provided comprehensive and reliable data on the occupational diseases diagnosed by physicians. The results suggested that the current situation of pneumoconiosis in Zhejiang Province was still serious, and government regulators should pay attention and further strengthen the supervision of industries in urgent. Moreover, publicity and education regarding pneumoconiosis should be carried out for dust workers to raise the awareness of dust exposure risk and associated health consequences.

There were some limitations couldn’t be neglected. Firstly, our data were based on the National Occupational Disease and Occupational Health Information Monitoring System reported by the occupational diseases diagnosis institution. In general, there existed delays and omissions in reporting [27], though China has already established reporting rules that reported in the system after diagnosis of occupational disease in 15 days. Secondly, some workers may not seek professional medical care due to some subjective reasons, which can also contribute to the under-reporting of pneumoconiosis cases. In addition, our study failed to get information on tuberculosis complications. Given that pneumoconiosis cases often present complications with some other diseases such as tuberculosis [28, 29], further studies with complication data should be conducted to explore the influence of pneumoconiosis category and exposure duration on the incidence of tuberculosis.

## Conclusion

Present study assessed the characteristics of pneumoconiosis in Zhejiang Province from 2006 to 2020. The findings suggested that the morbidity of pneumoconiosis was still severe in Zhejiang Province. Our study provides insight into the current status of pneumoconiosis and highlights the need for effective pneumoconiosis control strategies to prevent pneumoconiosis and protect workers’ health.

## Electronic supplementary material

Below is the link to the electronic supplementary material.

Supplementary Material 1

## Data Availability

The datasets used and analyzed during the current study are available from the corresponding author on reasonable request.

## Abbreviations

CWP: Coal workers’ pneumoconiosis
GDP: Gross domestic product
CDC: Centers for disease control and prevention
SD: Standard deviation
IQR: Interquartile range
