# Supplementary material for: Characteristics of pneumoconiosis in Zhejiang Province, China from 2006 to 2020: a descriptive study
Source: BMC Public Health. 2023 Feb 22;23:378. doi: 10.1186/s12889-023-15277-8 (PMC9948475; doi:10.1186/s12889-023-15277-8)
Supplement: Supplementary file 1 — Supplementary Material 1 [file 12889_2023_15277_MOESM1_ESM.docx]

**Characteristics of pneumoconiosis in Zhejiang Province, China from 2006 to 2020: A descriptive study**

**Fang Wei^1^, Panqi Xue^1^, Lifang Zhou^1^, Xinglin Fang^1^, Yixin Zhang^2^, Yong Hu^1^, Hua Zou^1*^, Xiaoming Lou^1*^**

^1^ Occupational Health and Radiation Protection Institute, Zhejiang Provincial Center for Disease Control and Prevention, Hangzhou, China.

^2^ School of Medicine, Hangzhou Normal University, China

**Table S1** The regional distribution of pneumoconiosis cases in Zhejiang Province reported from 2006 to 2020

| Region | 2006 | 2007 | 2008 | 2009 | 2010 | 2011 | 2012 | 2013 | 2014 | 2015 | 2016 | 2017 | 2018 | 2019 | 2020 | Total |
| --- | --- | --- | --- | --- | --- | --- | --- | --- | --- | --- | --- | --- | --- | --- | --- | --- |
| Taizhou | 7 | 1 | 59 | 61 | 237 | 4 | 182 | 204 | 7 | 14 | 14 | 17 | 5 | 190 | 178 | 1180 |
| Quzhou | 6 | 22 | 14 | 17 | 18 | 16 | 36 | 74 | 220 | 192 | 113 | 80 | 38 | 74 | 36 | 956 |
| Hangzhou | 5 | 42 | 32 | 35 | 76 | 82 | 160 | 160 | 106 | 60 | 38 | 50 | 30 | 27 | 24 | 927 |
| Ningbo | 80 | 46 | 41 | 29 | 29 | 45 | 46 | 62 | 170 | 59 | 65 | 39 | 46 | 29 | 27 | 813 |
| Huzhou | 26 | 12 | 7 | 21 | 63 | 79 | 90 | 65 | 61 | 32 | 35 | 75 | 58 | 43 | 20 | 687 |
| Wenzhou | 22 | 38 | 41 | 81 | 26 | 22 | 30 | 24 | 78 | 56 | 62 | 58 | 30 | 62 | 32 | 662 |
| Lishui | 0 | 5 | 5 | 21 | 13 | 18 | 19 | 11 | 15 | 13 | 17 | 25 | 23 | 20 | 19 | 224 |
| Jinhua | 3 | 4 | 5 | 9 | 10 | 22 | 13 | 16 | 8 | 11 | 7 | 20 | 51 | 11 | 9 | 199 |
| Jiaxing | 8 | 3 | 1 | 4 | 5 | 8 | 11 | 13 | 18 | 12 | 11 | 16 | 13 | 18 | 9 | 150 |
| Shaoxing | 0 | 0 | 4 | 1 | 4 | 1 | 20 | 9 | 16 | 15 | 10 | 7 | 14 | 18 | 6 | 125 |
| Zhoushan | 0 | 0 | 0 | 0 | 1 | 2 | 11 | 70 | 4 | 4 | 6 | 8 | 1 | 3 | 4 | 114 |
| Total | 157 | 173 | 209 | 279 | 482 | 299 | 618 | 708 | 703 | 468 | 378 | 395 | 309 | 495 | 364 | 6037 |

**Table S2** The post-hoc comparison results for diagnosis age and exposure duration

| Multiple comparison | *P* value | |
| --- | --- | --- |
|  | Diagnosis age | Exposure duration |
| Silicosis-CWP | <0.001* | 0.006* |
| Silicosis- Welders’ pneumoconiosis | <0.001* | <0.001* |
| Silicosis- Others | 0.106 | 0.706 |
| CWP- Welders’ pneumoconiosis | <0.001* | <0.001* |
| CWP - Others | <0.001* | 0.022* |
| Welders’ pneumoconiosis- Others | <0.001* | 0.002* |
| Stage I- Stage II | 0.808 | 0.282 |
| Stage I- Stage III | <0.001* | 0.010* |
| Stage II- Stage III | <0.001* | 0.499 |
| ‘<1980’ – ‘1980-’ | <0.001* | <0.001* |
| ‘<1980’ – ‘1990-’ | <0.001* | 0.999 |
| ‘<1980’ – ‘2000-’ | <0.001* | <0.001* |
| ‘<1980’ – ‘2010-’ | <0.001* | <0.001* |
| ‘1980-’ – ‘1990-’ | <0.001* | <0.001* |
| ‘1980-’ – ‘2000-’ | <0.001* | <0.001* |
| ‘1980-’ – ‘2010-’ | <0.001* | <0.001* |
| ‘<1990’ – ‘2000-’ | <0.001* | <0.001* |
| ‘<1990’ – ‘2010-’ | 0.008* | <0.001* |
| ‘<2000’ – ‘2010-’ | 0.378 | <0.001* |
| Mining - Manufacturing | <0.001* | <0.001* |
| Mining - Construction | 0.633 | <0.001* |
| Mining - Traffic, storage and mail business | <0.001* | 0.071 |
| Mining - Neighborhood services and other service industry | <0.001* | 0.118 |
| Mining - Public administration and social organization | <0.001* | <0.001* |
| Mining - Others | <0.001* | <0.001* |
| Manufacturing - Construction | <0.001* | 0.999 |
| Manufacturing - Traffic, storage and mail business | <0.001* | 0.886 |
| Manufacturing - Neighborhood services and other service industry | <0.001* | 0.999 |
| Manufacturing - Public administration and social organization | <0.001* | 0.815 |
| Manufacturing - Others | <0.001* | <0.001* |
| Construction - Traffic, storage and mail business | <0.001* | 0.979 |
| Construction - Neighborhood services and other service industry | <0.001* | 0.999 |
| Construction - Public administration and social organization | <0.001* | 0.999 |
| Construction - Others | <0.001* | 0.004* |
| Traffic, storage and mail business - Neighborhood services and other service industry | 0.999 | 0.999 |
| Traffic, storage and mail business - Public administration and social organization | 0.201 | 0.999 |
| Traffic, storage and mail business - Others | 0.515 | <0.001* |
| Neighborhood services and other service industry - Public administration and social organization | 0.457 | 0.999 |
| Neighborhood services and other service industry - Others | 0.642 | 0.013* |
| Public administration and social organization - Others | 0.999 | <0.001* |
| Large -Medium | <0.001* | <0.001* |
| Large -Small | 0.003* | <0.001* |
| Large -Micro | <0.001* | <0.001* |
| Large -Unknown | 0.105 | <0.001* |
| Medium -Small | <0.001* | 0.136 |
| Medium -Micro | 0.858 | 0.002* |
| Medium -Unknown | <0.001* | 0.177 |
| Small -Micro | 0.017* | 0.007* |
| Small -Unknown | 0.956 | 0.939 |
| Micro -Unknown | 0.013* | 0.015* |

*: *P*<0.05


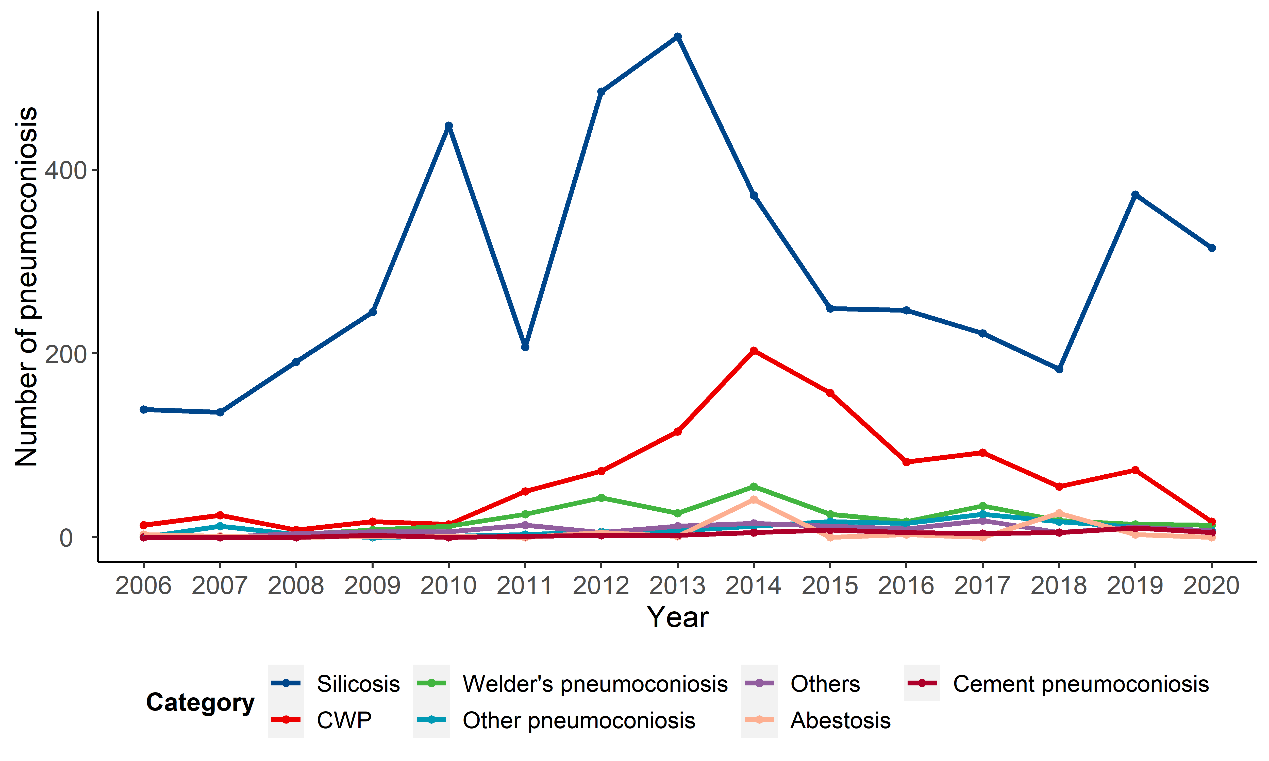


**Figure S1** The number of different categories of pneumoconiosis cases reported in Zhejiang Province from 2006 to 2020.


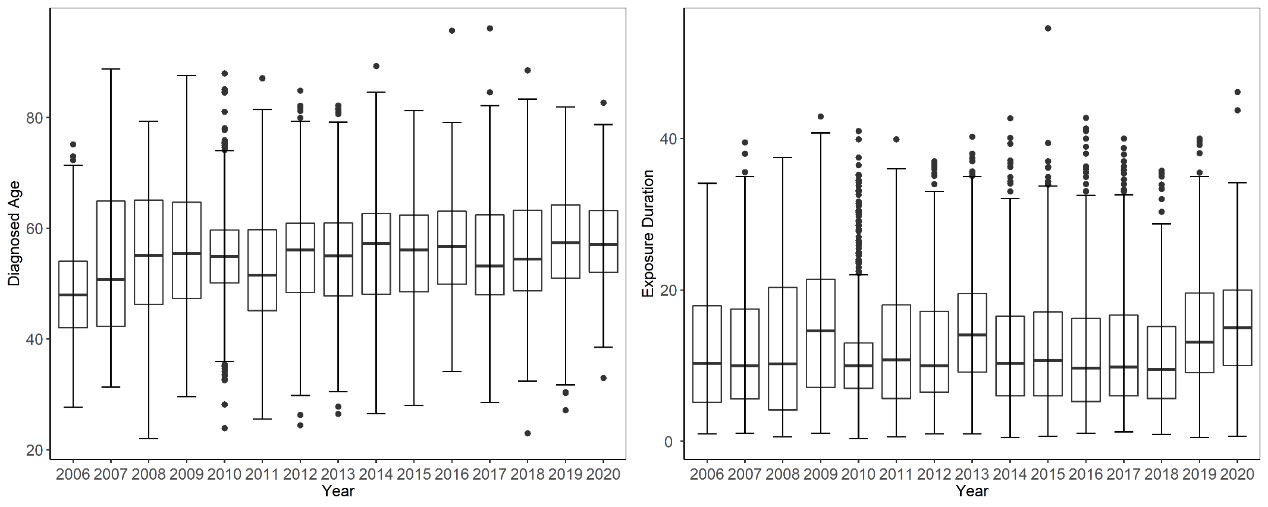


**Figure S2** The annual trend of diagnosis age and exposure duration of pneumoconiosis cases in Zhejiang Province from 2006 to 2020
